# Early life stress in women with autoimmune thyroid disorders

**DOI:** 10.1038/s41598-023-49993-3

**Published:** 2023-12-15

**Authors:** Alessia Corso, Hermann Engel, Fabienne Müller, Serena Fiacco, Laura Mernone, Elena Gardini, Ulrike Ehlert, Susanne Fischer

**Affiliations:** 1 Institute of Psychology, Clinical Psychology and Psychotherapy, University of Zurich, Binzmuehlestrasse 14, Box 26, 8050 Zurich, Switzerland; 2 Thyroid Center Zurich, Zurich, Switzerland; 3 URPP Dynamics of Healthy Aging Research Priority Program, University of Zurich, Zurich, Switzerland

**Keywords:** Thyroid diseases, Risk factors

## Abstract

Autoimmune thyroid disorders (AITD) represent the most frequent of all autoimmune disorders. Their aetiopathogenesis is incompletely understood, but most likely multifactorial. Early life stress can have long-lasting effects on the immune system. The aim of the present study was to investigate, for the first time, whether patients with AITD are more frequently affected by early life stress. A total of N = 208 women were recruited into a case–control study. Of these, *n* = 78 (median age: 53, interquartile range: 15) were patients recruited from a thyroid outpatient clinic with confirmed Hashimoto’s thyroiditis, Graves’ disease, or AITD not otherwise specified. The remaining *n* = 130 age- and BMI-matched women (median age: 53, interquartile range: 12) were recruited from the general population. Early life stress was measured with the Childhood Trauma Questionnaire. Patients with AITD did not differ from controls regarding sexual abuse, physical abuse, and physical neglect. However, a greater number of patients reported emotional neglect (29.7% vs. 19.5%) and emotional abuse (41.3% vs. 32%). This study provides initial evidence for emotional neglect and abuse as potential risk factors for the development of AITD. Prospective confirmation of these findings could pave the way for the development of interventions to prevent AITD in predisposed individuals.

## Introduction

Autoimmune thyroid disorders (AITD) are the most frequent of all autoimmune disorders^1,2^, with Hashimoto’s thyroiditis and Graves’ disease representing its most severe forms^3,4^. The hypothalamic-pituitary-thyroid is regulated at multiple levels, including the release of hypothalamic thyrotropin-releasing hormone from the hypothalamus, thyroid stimulating hormone from the pituitary, and the thyroid hormones triiodothyronine and thyroxine from the thyroid gland. Moreover, the activation of thyroid hormones is governed by deiodinases, with type II deiodinase converting inactive thyroxine into active triiodothyronine. Both Hashimoto’s thyroiditis and Graves’ disease present with lymphocytic infiltration of the thyroid gland^1,5,6^. Hashimoto’s thyroiditis is predominantly characterised by a T cell-mediated immune response leading to thyroid destruction and, consequently, often presents with hypothyroidism^4–7^. Additionally, thyroid autoantibodies against thyroid peroxidase and thyroglobulin are often detected^5,6,8,9^. By contrast, Graves’ disease is primarily characterised by an antibody-mediated immune response leading to thyroid hormone overproduction. This causes hyperthyroidism due to the presence of thyroid-stimulating hormone receptor antibodies^4,10–12^. Older individuals are more frequently affected by AITD^3^ and women have a seven to eighteen times higher risk of developing AITD^2,3^. Given that AITD are chronic diseases that often require lifelong medication^13,14^, it is important to identify risk factors for their prevention.

Although the mechanisms that trigger autoimmunity against the thyroid are unclear, the aetiopathogenesis of AITD is, presumably, multifactorial^1,9,15–17^. Twin studies have confirmed the role of genetic factors in AITD^18–20^, with heritability estimates between 70 and 80%^16,19^. In addition, several environmental risk factors have been identified, including smoking, viral and bacterial infections, iodine intake, medication^1,9,16,17,21^, as well as vitamin D and selenium deficiency^9^, different environmental pollutants^17,21^ and external and internal irradiation^1,17^. Furthermore, there is increasing evidence that psychosocial stress is associated with the development of AITD^17,22^. Whereas the role of stress in the onset of Graves’ disease has been confirmed by numerous studies^4,22^, much less is known about a potential relationship between stress and Hashimoto’s thyroiditis. Importantly, similar to depression^23,24^, accumulating research not only suggests a relationship between stress in adulthood and AITD, but also between early life stress and AITD^25–30^.

In line with this notion, there is long-standing evidence that early life stress leads to altered immune functioning. This is presumably mediated by the two major stress-responsive systems, the sympathetic nervous system and the hypothalamic–pituitary–adrenal axis^31,32^. Both systems engage in a crosstalk with the immune system, and in manifold ways. For instance, catecholamines and glucocorticoids, the end products of these two systems, have been shown to induce a shift in the Th1/Th2 balance and release of pro- and anti-inflammatory cytokines, which may constitute a possible explanation for the development of AITD^22,33,34^. In line with this, numerous studies have found an association between early life stress and chronic low-grade inflammation in adulthood, both in healthy samples^25–27^ and in individuals with depression^35–37^. Furthermore, early life stress also seems to have a direct effect on the hypothalamic-pituitary-thyroid axis. For instance, elevated levels of thyroid-stimulating hormone have been observed in women with childhood sexual abuse^28^. Finally, individuals exposed to childhood trauma had an increased risk of developing autoimmune disorders^29^ and of developing thyroid disorders^30^.

In sum, there is considerable evidence which suggests that early life stress is involved in the aetiopathogenesis of AITD. However, to date, no studies have examined this potential link. The aim of the present case–control study was thus to investigate whether patients with AITD and healthy controls differ in the amount of experienced early life stress. It was hypothesised that patients with AITD would have higher rates of early life stress than healthy controls.

## Results

### Participant characteristics

Sociodemographic and lifestyle characteristics of the patients and the healthy controls are reported in Table 1. On average, the women in the sample were of middle age (median age: 53, interquartile range: 13), had a vocational education or a college/university degree (80%), were mothers of two children (interquartile range: 2), had a normal Body Mass Index (median: 22, interquartile range: 4; 26% were overweight and 5% were obese), and were non-smokers (87%). The patients and the controls did not differ in any of the sociodemographic and lifestyle variables, except for menopausal status, with more patients being in the perimenopausal phase (statistical trend).

**Table 1:** Sociodemographic and lifestyle characteristics of patients with autoimmune thyroid diseases (AITD) and healthy controls.

	Patients (*n* = 78)		Healthy controls (*n* = 130)		*p*
	*n* (%)	*Mdn* (*IQR*)	*n *(%)	*Mdn *(*IQR*)	
Age (years)		53 (15)		53 (12)	0.18
BMI		23 (5)		22 (4)	0.40
Children (number)		2 (2)		2 (2)	0.61
Smoking (yes)	12 (15)		15 (12)		0.42
Menopause status*					0.08
Pre	30 (39)		56 (43)		
Peri	14 (18)		10 (8)		
Post	33 (43)		64 (49)		
AITD					
Hashimoto’s thyroiditis	52 (67)				
Graves’ disease	8 (10)				
Not otherwise specified	18 (23)				

Group comparisons were conducted using two-sided Mann–Whitney U tests and Pearson’s Chi-squared tests.

*Mdn* median, *IQR* interquartile range, *BMI* body mass index, *AITD* autoimmune thyroid diseases.

*Patients were categorised by self-report, whereas healthy controls were categorised by the Stages of Reproductive Aging Workshop + 10 (STRAW) criteria^56^: (1) pre if the menstrual cycle was regular, (2) peri if the cycle length was variable > 7 days or interval between cycles > 60 days, and (3) if there was no bleeding within 12 months.

Within the patient group, 67% of the women were diagnosed with Hashimoto’s thyroiditis (E.06.3), 10% with Graves’ disease (E05.0), and 23% with an AITD not otherwise specified (E06.9). The average age at diagnosis was 51 ± 11 and 54% had a family history of AITD. Two thirds of the patients were currently taking thyroid medication (e.g., l-thyrox, Euthyrox, Eltroxin) and 72% were euthyroid. With regard to comorbid diseases, 13% of the patients had a cardiovascular disease, 4% had an inflammatory disease, 4% had a metabolic disease, 6% suffered from recurrent headaches and 12% suffered from an affective disorder.

### Early life stress in patients with autoimmune thyroid disorders versus healthy controls

**Figure 1:**
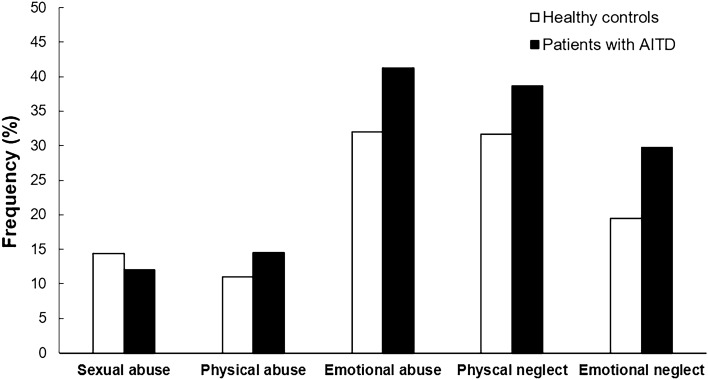
Frequencies of childhood trauma (Childhood Trauma Questionnaire; CTQ) in patients with autoimmune thyroid disorders (AITD) and healthy controls. Group comparisons were conducted using one-sided Pearson’s Chi-squared tests. A greater proportion of patients reported emotional abuse (41.3% vs. 32%; Χ^2^ = 1.779, *p* = 0.091, *w* = 0.092) and emotional neglect (29.7% vs. 19.5%; Χ^2^ = 2.695, *p* = 0.051, *w* = 0.114).

As is evident from Fig. 1, patients with AITD did not differ from healthy controls in terms of moderate to severe trauma regarding sexual abuse (12% vs. 14.4%; Χ2 = 0.231, *p* = 0.32), physical abuse (14.5% vs. 11%; Χ2 = 0.524, *p* = 0.24), and physical neglect (38.7% vs. 31.7%; Χ2 = 1.003, *p* = 0.16). However, a greater proportion of patients reported emotional abuse (41.3% vs. 32%; Χ2 = 1.779, *p* = 0.091, *w* = 0.092) and emotional neglect (29.7% vs. 19.5%; Χ2 = 2.695, *p* = 0.051, *w* = 0.114).

## Discussion

The main finding of this study is that female patients with AITD tended to have a higher frequency of emotional abuse and emotional neglect when compared to healthy controls. There were no differences between the two groups regarding other forms of childhood trauma. These findings will be discussed in the following paragraphs.

This is the first study to examine childhood trauma in AITD. Compared to a norm sample of German women of similar age^38^, the prevalence of emotional neglect in women with AITD was more than twice as high (29.7% vs. 13.4%) and the prevalence of emotional abuse was more than three times as high (41.3% vs. 10.9%), while the other forms did not show such marked differences. This suggests that emotional forms of childhood trauma may be particularly important risk factors for AITD. Notably, the prevalence of emotional neglect was also slightly higher in the healthy control group when compared to the norm sample (19.5% vs. 13.4%) and this was even more pronounced for emotional abuse (32% vs. 10.9%)^38^.

Our finding is in line with previous research showing that less obvious forms of maltreatment, such as emotional abuse and neglect, can have equally significant consequences for individuals’ development and health^39^. Childhood neglect has indeed been found to have long-term effects on children’s cognitive, social-emotional and behavioural development^40^. Moreover, it has also been demonstrated to affected children’s neuroendocrine stress response system up into adulthood^41–43^. Interestingly, there is evidence that different childhood trauma types lead to different changes in the hypothalamic–pituitary–adrenal axis and in the immune system^44^. In a meta-analysis of this literature, sexual and physical abuse were significantly associated with elevated tumour necrosis factor α and interleukin 6 concentrations, whereas neglect was primarily associated with elevated C-reactive protein levels^45^. It thus appears as if different types of childhood trauma were associated with different health outcomes. Interestingly, one study found elevated C-reactive protein levels in individuals with various types of hypo- and hyperthyroidism^46^, although other studies in Hashimoto thyroiditis or Graves’ disease could not confirm this association^47,48^. The jury is thus still out on whether long-lasting effects of emotional early life stress on immune functioning may facilitate the onset of AITD in combination with genetic and other environmental factors (e.g., stress in adulthood).

This study presents with a number of strengths. First and foremost, it is the first study investigating the association between early life stress and AITD. To this end, a state-of-the-art instrument was used to assess childhood trauma in five domains. However, a number of limitations should also be mentioned. First, the patient sample size was relatively small and smaller than the healthy control sample, which may have contributed to the borderline significant findings. Related to this, it was not possible to undertake group comparisons between patients with Hashimoto’s thyroiditis and Graves’ disease. Further, large-scale studies which are specifically designed for such case–control comparisons are thus warranted. Second, the female patient group in this study means that the results cannot be generalised to the male population and to individuals of other genders. Although, based on the extant literature, we do not expect that the relationship between early life stress and autoimmunity differs according to gender^25–27,29^, the present findings warrant replication in samples of non-female patients with AITD. Third, due to the lack of laboratory testing in the control group, inflammatory markers, thyroid parameters, and related antibodies could not be assessed. Therefore, potentially ongoing inflammatory processes and the presence of subclinical hypo-/hyperthyroidism and elevated antibodies in the control group cannot be excluded and may have obfuscated any further group differences. However, it should be mentioned that, due to the strict inclusion criteria, the control group had a particularly high health status. Finally, the retrospective study design and the fact that we used a self-reported instrument to assess childhood trauma does not allow to exclude potential memory biases regarding the reporting of childhood trauma.

In sum, the present study provides initial, tentative evidence for emotional early life stress as a potential risk factor for the development of AITD. Measures mitigating the detrimental effects of emotional early life stress may thus be considered to prevent the development of AITD. These could target individuals with family histories of autoimmune disorders who report early life stress, but have not yet developed AITD. Similar to other lifestyle variables which constitute risk factors for AITD, this could mean that predisposed individuals are advised to actively manage their stress (e.g., by efficacious programmes such as mindfulness-based stress reduction). This could not only have beneficial effects on the immune system^49^ and, potentially, on the hypothalamic-pituitary-thyroid axis^50^, but also help to prevent depression and anxiety, which frequently feature in these conditions^51,52^. However, further, large-scale cohort studies are necessary to replicate and extend the herein reported findings before such measures could potentially be considered.

## Methods

### Participants and protocol

A total of N = 208 women were recruited into a case–control study. Of these,* n* = 78 were patients with AITD. The patients were recruited at the Thyroid Center Zurich, which constitutes the largest outpatient clinic exclusively specialising in thyroid diseases in Switzerland. Autoimmune thyroid disorders were diagnosed by a thyroid specialist (HE) and based on clinical features, laboratory testing (serum levels of thyroid-stimulating hormone, free triiodothyronine, and free thyroxine, as well as thyroglobulin antibodies, thyroid peroxidase antibodies and/or thyroid-stimulating hormone receptor antibodies), and sonographic morphology and perfusion. All patients who had a diagnosis of Hashimoto’s thyroiditis (E.06.3), Graves’ disease (E05.0), or AITD not otherwise specified (E06.9), which represented the bulk of AITD at the outpatient clinic, were asked whether they would be interested in taking part in the study. Further inclusionary criteria were being fluent in German and between 40 and 75 years of age. Patients who met these criteria were asked to fill in the Childhood Trauma Questionnaire, as detailed below. In addition, a total of *n* = 130 women from a study on healthy ageing were used as healthy controls. This sample has previously been described in detail^53^. In brief, women between the age of 40 and 75 who had to be free of any somatic diseases (including acute infections) or mental disorders were recruited via flyers and advertisements in newspapers and social media. Importantly, both samples were recruited during the same time period (i.e., from 2017 to 2020) and in the same geographical area (i.e., the Canton of Zurich).

Written informed consent was obtained for participation in both studies and the study protocol was approved by the Ethics Committee of the Canton of Zurich (BASEC 2021-00983). All methods were performed in accordance with the relevant guidelines and regulations.

### Psychological data

The short version of the Childhood Trauma Questionnaire^38,54^ was used to measure *early life stress*. The Childhood Trauma Questionnaire is one of the most frequently used psychometric instruments to assess early life stress worldwide. It has been extensively validate and also proven to be reliable (Cronbach’s alpha ≥ 0.89) and valid (e.g., regarding its factorial structure and correlation with trauma-associated disorders) in German-speaking samples^55^. It has 25 items assessed on a five-point Likert scale (from 1 to 5) and distinguishes five trauma domains consistent with the consensus definition of childhood trauma^38^: emotional neglect, physical neglect, emotional abuse, physical abuse, and sexual abuse. Example items are: “When I was growing up, people in my family looked out for each other” (emotional neglect, reversed item), “When I was growing up, my parents were too drunk or high to take care of the family” (physical neglect), “When I was growing up, people in my family called me things like *stupid*, *lazy*, or *ugly*” (emotional abuse), “When I was growing up, I was punished with a belt, a board, a cord, or some other hard object” (physical abuse), “When I was growing up, someone tried to touch me in a sexual way, or tried to make me touch them” (sexual abuse). The presence vs. absence of childhood trauma was determined based on validated cut-off scores for moderate to severe trauma^38^. These were ≥ 15 for emotional neglect, ≥ 8 for physical neglect, ≥ 10 for emotional abuse, ≥ 8 for physical abuse, and ≥ 8 for sexual abuse.

### Medical data

The medical data extracted from the clinic’s data base consisted of diagnoses, thyroid parameters (thyroid-stimulating hormone, free triiodothyronine, free thyroxine, antibodies), and information about thyroid medication intake.

### Laboratory analyses

Thyroid parameters (i.e., thyroid-stimulating hormone, free triiodothyronine, free thyroxine, antibodies) were determined in the biochemical laboratory at the Thyroid Center Zurich, which is an outpatient clinic located in a large hospital. Blood was collected by means of venepuncture and all samples were processed and analysed in loco before being entered into the data base.

### Statistical analyses

Assuming a medium effect size (α = 0.05, 1 − β = 0.80), an a priori power analysis resulted in a total sample size of *N* = 88. Due to deviation from a normal distribution and/or missing homoscedasticity, all continuous variables were analysed by means of Mann–Whitney U tests. Chi-squared tests were computed to compare patients and controls in terms of dichotomous variables. All analyses were conducted using the Statistical Package for the Social Sciences (SPSS) 25, and the main analyses were tested one-tailed with a significance level of α < 0.05.

## Data Availability

Data is not publicly available due to ethical reasons. Further enquiries can be directed to the corresponding author.
